# Maternal communication and attachment following a group singing intervention for postnatal depression: findings from the SHAPER-PND trial

**DOI:** 10.1017/S0033291726103997

**Published:** 2026-04-07

**Authors:** Lavinia Rebecchini, Rebecca H. Bind, Carolina Estevao, Katie Hazelgrove, Kristi Priestley, Riddhi Laijawala, Samrina Sangha, Vaheshta Sethna, Anthony J. Woods, Nikki Crane, Manonmani Manoharan, Alexandra Burton, Hannah Dye, Tim Osborn, Lorna Greenwood, Daisy Fancourt, Carmine M. Pariante, Paola Dazzan

**Affiliations:** 1 Department of Psychological Medicine, King’s College London, Institute of Psychiatry Psychology & Neuroscience (IoPPN), UK; 2 Social, Genetic & Developmental Psychiatry Centre, King’s College London, Institute of Psychiatry Psychology & Neuroscience (IoPPN), UK; 3 Culture Team, King’s College London, UK; 4 South London and Maudsley NHS Foundation Trust, UK; 5 Department of Behavioural Science and Health, University College London, UK; 6 Breathe Arts Health Research, The Clerance Centre, UK

**Keywords:** arts health interventions, group singing, maternal communication, mother–infant interaction, postnatal attachment, postnatal depression

## Abstract

**Background:**

Postnatal depression (PND) can disrupt maternal communication during early interactions, affecting infant socioemotional development. Singing is a natural form of caregiver–infant communication and a promising intervention to enhance maternal well-being and bonding. However, its effects on observed communication and perceived attachment in clinical PND populations remain underexplored.

**Methods:**

Within the Scaling-Up Health-Arts Programs: Postnatal Depression trial, 199 mothers with PND were randomized 2:1 to a 10-week group singing intervention (Breathe Melodies for Mums) or a non-singing community activity. One hundred participants (singing = 70; control = 30) completed video-recorded interactions at baseline, week 10, and week 36. Maternal speech was coded using the Parental Cognitive Attributions and Mentalization Scale (PCAMS) for mentalization, affective tone, and attentional focus. Perceived maternal attachment was assessed separately via self-report using the Maternal Postnatal Attachment Scale.

**Results:**

At week 10, singing mothers showed greater improvement in communication with their infants than controls, with about 1.7-fold higher proportions of mentalizing comments (*p* = 0.01), 1.4-fold more infant-focused speech (*p* < 0.001), 2.4-fold less parent-focused speech (*p* < 0.001), and fivefold less negative speech (*p* < 0.001). These effects were maintained at week 36. Perceived attachment improved significantly across both groups (*p* < 0.001), but only singing mothers showed further gains from week 10 to week 36 (*p* = 0.02), indicating continued strengthening of attachment perceptions.

**Conclusions:**

Group singing enhanced maternal communication and perceived attachment in mothers with PND. Findings support community-based, arts-informed interventions as accessible approaches to strengthen early relational health and complement perinatal mental healthcare.

## Introduction

Postnatal depression (PND) affects up to 20% of mothers worldwide and is associated with disruptions in mother–infant interaction, including reduced sensitivity, responsiveness, and communicative attunement (Murray & Cooper, 1997; Tronick & Reck, 2009; Wisner, Parry, & Piontek, 2002; Woody et al., 2017). These difficulties are clinically significant, as early relational exchanges lay the foundation for infants’ socioemotional, cognitive, and language development (Feldman, 2007). Importantly, treating maternal mood alone often fails to remediate impairments in maternal communication and interaction (Forman et al., 2007), underscoring the need for interventions that target both depressive symptoms and relational functioning.

Pharmacological and psychological treatments, including cognitive–behavioral therapy and interpersonal psychotherapy, are effective in reducing depressive symptoms (Cuijpers, Van Straten, Andersson, & Van Oppen, 2008; Sockol, 2018). Yet systematic reviews suggest that such approaches do not consistently improve maternal sensitivity or communication with the infant (Letourneau, Dennis, Cosic, & Linder, 2017). Consequently, there is increasing interest in community-based, scalable interventions that can simultaneously support maternal well-being and strengthen early relational health.

Arts-based programs represent a promising avenue. Group activities provide opportunities for social support and peer connection, which are protective factors against depression (Bickerdike et al., 2017). Singing has particular relevance, being embedded in the earliest forms of caregiver–infant communication. Research on communicative musicality highlights how vocal rhythm, melody, and affective vocalizations underpin synchrony, affect regulation, and joint attention in infancy (Malloch & Trevarthen, 2009; Trevarthen & Malloch, 2000). Maternal singing may therefore both extend natural communicative patterns and serve as a targeted strategy to enhance mother–infant bonding (Rebecchini, 2021).

Empirical studies support these dual benefits. Mothers who sing to their infants report enhanced bonding and more positive affect (Vlismas, Malloch, & Burnham, 2013), and experimental work demonstrates that singing reduces infant distress and fosters reciprocity (Coulton, Clift, Skingley, & Rodriguez, 2015; Nakata & Trehub, 2004). At the group level, structured singing interventions have been shown to reduce depressive symptoms in mothers with PND (Fancourt & Perkins, 2018). However, rigorous evaluation of their effects on observed maternal communication and perceived bonding toward their infants, particularly in clinically depressed populations, remains scarce.

The Scaling-Up Health-Arts Programs: Postnatal Depression (SHAPER-PND) trial was designed to evaluate Breathe Melodies for Mums, a structured, 10-week group singing program for mothers with PND, delivered at scale in community settings (Estevao et al., 2021a, 2021b). The primary outcome paper demonstrated significant reductions in depressive symptoms in the singing group compared with the non-singing community activity, but the differences between the groups became evident 10 weeks after the end of the interventions and were sustained 6 months after the intervention (Bind et al., 2025). Alongside these primary outcomes, sub-studies were developed to examine potential mechanisms of change, including maternal communication and attachment.

### Current study

The present sub-study is the first to investigate whether the benefits of group singing extend to relational outcomes both during (baseline and 10 weeks) and 6 months after the intervention. Drawing on observational and self-report measures, we examine maternal communication – operationalized as mentalizing comments, attentional focus, and affective state of speech – and maternal perceptions of attachment over the intervention and follow-up period. By situating this work within the SHAPER-PND trial, we provide novel evidence on the relational impact of singing interventions, advancing the case for scalable, arts-informed strategies that support both maternal recovery and early parent–infant bonding.

## Methods

### Study design and participants

This study analyzed data from a subsample of the SHAPER-PND study (Bind et al., 2025), a two-arm, effectiveness–implementation hybrid type 2 trial evaluating the impact, for mothers with PND, of a specially designed group singing program, Breathe Melodies for Mums (M4M), compared with participation in usual community activities that did not involve singing. The intervention was also piloted online during the COVID-19 period (Bind et al., 2023). The present study focused on participants who completed the video-recorded assessments of mother–infant interaction at baseline, week 10, and week 36.

In the main trial, a total of 199 mothers with infants aged 0–9 months were enrolled and randomized (133 to singing, 66 to control). Eligibility required a score of ≥10 on the Edinburgh Postnatal Depression Scale (EPDS; Cox, Holden, & Sagovsky, 1987), indicating clinically relevant depressive symptoms. Additional inclusion criteria were age ≥ 18 years, sufficient English proficiency, and access to an internet-connected device. Exclusion criteria included an EPDS score < 10, inability to provide informed consent, and participation in concurrent singing or music-based programs.

Of the 199 participants, 185 (93%) provided consent for video recording (singing *n* = 122; control *n* = 63). At baseline, 160 dyads (singing *n* = 112; control *n* = 48) completed video-recorded interactions. At week 10, 120 dyads were recorded (singing *n* = 88; control *n* = 32), and at week 36, 90 dyads were recorded (singing *n* = 72; control *n* = 18). Following exclusions for poor quality recordings, foreign language use, twin status, or missing paired data, 100 participants (singing *n* = 70; control *n* = 30) had analyzable baseline and week 10 videos, and 67 participants (singing *n* = 54; control *n* = 13) had analyzable videos at all three timepoints. Furthermore, a total of 92 participants completed self-report measure of perceived attachment at the three timepoints (singing *n* = 64; control *n* = 28). Reasons for exclusion are detailed in Supplementary Figure S1. Comparisons between included participants and those without available video data are presented in Supplementary Table S1.

### Randomization and procedures

Participants were randomized 2:1 (singing: control) stratified by baseline EPDS and infant age; full details are provided in Supplementary Material, Methods section.

Participants allocated to the intervention attended the Breathe Melodies for Mums (M4M) program, consisting of 10 weekly, hour-long group singing sessions delivered in community children’s centers across South London. A detailed description of the intervention can also be found in the study protocol (Estevao et al., 2021a) as well as the main outcomes paper (Bind et al., 2025).

Control group participants were signposted to other non-music mother–infant activities available in the community (e.g. baby massage and messy play) and received the same schedule of reminders as the singing intervention group to encourage attendance. Following the 10-week study period, control participants were offered the opportunity to join the next available singing group outside the intervention period.

Session attendance for the singing intervention was recorded for program management purposes; however, attendance rates were not formally analyzed as predictors of outcome. The trial followed an intention-to-treat design (Bind et al., 2025), evaluating the effect of allocation to the singing intervention versus active control. Attendance at community activities in the control group was not systematically monitored.

### Measures

#### Sociodemographic and clinical measures

Sociodemographic and socioeconomic (SES) variables were ascertained with a semi-structured interview. A composite SES score was derived from baseline sociodemographic variables following established cumulative risk approaches (see Supplementary Methods). Depressive symptoms were assessed with the Edinburgh Postnatal Depression Scale (EPDS; Cox et al., 1987), which rates symptoms on 4-point Likert scale (0–3), yielding a total score ranging from 0 to 30. Higher scores indicate greater symptom severity. Consistent with validation studies (Cox et al., 1987; Matthey, Henshaw, Elliott, & Barnett, 2006), a threshold of ≥10 was applied to identify women at risk of PND.

#### Maternal communication during observed mother–infant interaction

Mother–infant interactions were video recorded at baseline, week 10, and week 36. Dyads were instructed to engage in free play for three minutes. Maternal speech was transcribed verbatim and coded using the Parental Cognitive Attributions and Mentalization Scale (PCAMs; Rebecchini et al., 2023; Sethna, Murray, & Ramchandani, 2012. The PCAMs quantifies maternal communication across three domains: (i) mentalizing comments (awareness and interpretation of infants’ cognitive, emotional, and physiological states); (ii) attentional focus (infant-, parent-, or other-focused speech); and (iii) affective connotation (positive or negative comments). For each domain, raw scores were first calculated as the total number of utterances falling into that category; these were then expressed as proportions relative to the total number of utterances for each participant, thereby controlling for individual variability in overall speech output. Accordingly, all comparative ratios reported in the Results refer to differences in these proportional values rather than to raw frequency counts. Videos were coded blind to group allocation. The coding manual is available from the authors upon request. Inter-rater reliability and examples of maternal comments and their ratings are presented in the Supplementary Materials (Methods).

#### Maternal perceived bonding toward the infant

Perceived maternal attachment was assessed at baseline, week 10, and week 36 using the Maternal Postnatal Attachment Scale (MPAS; Condon & Corkindale, 1998, a 19-item self-report measure of the mother–infant emotional bond. In the present analyses, we used the MPAS total (global) score as an overall index of perceived attachment. Throughout the manuscript, the terms ‘perceived attachment’ and ‘global attachment’ refer to this total score. Further details regarding subscales and scoring are provided in the Supplementary Materials.

### Statistical analysis

Analyses were conducted using IBM SPSS Statistics (Version 31). Continuous variables are reported as mean (SD) and categorical variables as frequency (%). Baseline group differences were examined using independent samples t-tests, Mann–Whitney U tests, or chi-square tests as appropriate.

Two-way repeated measures ANOVAs were used to examine group (singing vs. control) × time (baseline, week 10, week 36) effects for PCAMs proportional speech variables and MPAS global attachment scores. Significant interactions were followed by planned contrasts. Effect sizes are reported as partial eta squared (*η* ^2^).

Pearson’s correlations were performed to explore associations between SES, EPDS scores, and communication variables demonstrating significant group effects. Statistical significance was set at *p* < .05 (two-tailed).

Further details regarding assumption testing, sphericity corrections, sensitivity analyses, and missing data handling are provided in the Supplementary Materials.

## Results

### Sociodemographic and clinical characteristics

Sociodemographic characteristics of the participants are presented in Table 1.

**Table 1.** Sociodemographic, clinical, and infant-related characteristics

	Control group (*n* = 19–30)	Singing group (*n* = 61–70)	Statistic
Maternal age in years, M (SD)	34.6 (4.8)	35.7 (4.0)	*p* = 0.25
Maternal ethnicity, *n* (%) Any white background Ethnic minority groups	21 (70%) 9 (30%)	50 (71.4%) 20 (28.6%)	*p* = 0.89
Maternal qualifications, *n* (%) Higher education or above GCSE/A level or below	27 (90%) 3 (10%)	63 (90%) 7 (10%)	*p* = 1.00
Maternal employment status, *n* (%) Employed/student/maternity leave Unemployed/full-time mother	27 (90%) 3 (10%)	63 (90%) 7 (10%)	*p* = 1.00
Household income, *n* (%) <£30,000 ≥£30,000	7 (25.9%) 20 (74.1%)	9 (13.2%) 59 (86.8%)	*p* = 0.14
Maternal marital status, *n* (%) Married/cohabiting Single with/without a partner	21(70%) 9 (30%)	60 (85.7%) 10 (14.3%)	*p* = 0.07
SES score, M (SD)	0.72 (0.2)	0.76 (0.2)	*p* = 0.41
Infant sex, *n* (%) Female Male	15 (50.0%) 15 (50.0%)	31 (44.9%) 38 (55.1%)	*p* = 0.65
Attending other mother-baby group, *n* (%) Yes No	20 (66.7%) 10 (33.3%)	51 (72.9%) 19 (27.1%)	*p* = 0.57
Currently receiving any type of psychological therapy, *n* (%) Yes No	10 (33.3%) 20 (66.7%)	25 (35.7%) 45 (64.3%)	*p* = 0.82
Infant age at baseline in months, M (SD)	4.2 (2.5)	4.1 (2.4)	*p* = 0.80
Infant age at week 10 in months, M (SD)	7 (2.8)	6.6 (2.4)	*p* = 0.54
Infant age at week 36 in months, M (SD)	12.6 (2.9)	12.3 (2.4)	*p* = 0.73
EPDS baseline score, M (SD)	16.2 (4.2)	16.5 (3.8)	*p* = 0.73
EPDS week 10 score, M (SD)	10.7 (4.3)	10.2 (4.6)	*p* = 0.66
EPDS week 36 score, M (SD)	8.4 (4.4)	8.0 (4.5)	*p* = 0.70

*Note*: SES, sociodemographic and socioeconomic score; EPDS, Edinburgh Postnatal Depression Scale.

As expected with a randomization process, there were no statistically significant group differences in maternal age (*p* = 0.25), ethnicity (*p* = 0.89), education (*p* = 1.00), employment status (*p* = 1.00), marital status (*p* = 0.07), or annual income (*p* = 0.14). There were also no significant differences in SES scores between women in the control (0.73 ± 0.2) and singing group (0.78 ± 0.2) (*p* = 0.24).

### Impact of M4M on maternal communication and perceived attachment to their infant

#### Quality of maternal communication was significantly enhanced in the singing group compared with the control group at the end of the intervention

**Figure 1.**
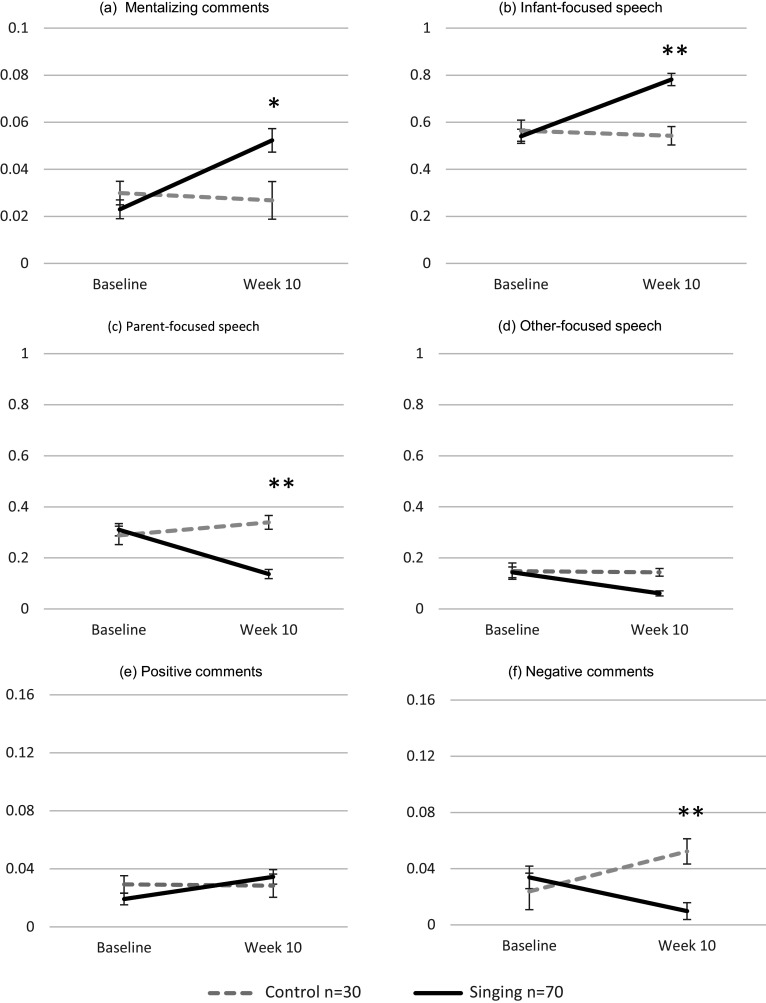
Mean proportion scores for all coded maternal speech domains in the singing and control groups across baseline and week 10. Panels (a–f) show mentalizing comments, infant-focused speech, parent-focused speech, other-focused speech, positive comments, and negative comments, respectively (*p < 0.05; **p < 0.01).

Results for differences in the proportions of speech domains between the control and singing groups at baseline and week 10 are presented in Figure 1, with raw data shown for the 100 women who completed both video assessments. Results on main effects of time are reported in the Supplementary Materials (Results).

For mentalizing comments (Figure 1a), a significant group × time interaction showed that the rate of change differed between groups (*F*(1, 98) = 10.46, *p* = 0.002, partial *η* ^2^ = 0.096). At week 10, the proportion of mentalizing comments was approximately 1.7 times higher in the singing group compared with controls (mean difference = −0.026 [95% CI –0.044, −0.007], *F*(1, 98) = 7.64, *p* = 0.01, partial *η* ^2^ = 0.072), while no difference was found at baseline. This indicates that mothers in the singing group used a greater proportion of infant mental state-related language during the observed interaction.

For infant-focused speech (Figure 1b), there was also a significant group × time interaction (*F*(1, 98) = 18.46, *p* < 0.001, partial *η* ^2^ = 0.159). At week 10, mothers in the singing group used about 1.4 times more infant-focused comments than controls (mean difference = −0.239 [95% CI –0.332, −0.147], *F*(1, 98) = 26.07, *p* < 0.001, partial *η* ^2^ = 0.210), with no baseline differences, suggesting that the intervention fostered a more infant-focused orientation in maternal communication, reflecting greater emphasis on the infant’s perspective during interaction.

For parent-focused speech (Figure 1c), a significant group × time interaction confirmed diverging trajectories across groups (*F*(1, 98) = 21.29, *p* < 0.001, partial *η* ^2^ = 0.178). By week 10, parent-focused speech was roughly 2.4 times lower in the singing group than in the control group (mean difference = 0.204 [95% CI 0.139, 0.269], *F*(1, 98) = 38.21, *p* < 0.001, partial *η* ^2^ = 0.281), while no difference was observed at baseline, indicating a reduction in speech centered on the mother’s own agenda or perspective during interaction.

For other-focused speech (Figure 1d), the group × time interaction was nonsignificant (*F*(1, 98) = 0.64, *p* = 0.43, partial *η* ^2^ = 0.006), suggesting similar decreases in both groups. This overall reduction in comments unrelated to the play context implies that mothers became increasingly focused on the ongoing interaction with their infants.

For positive comments (Figure 1e), no significant group × time interaction emerged (*F*(1, 98) = 2.29, *p* = 0.13, partial *η* ^2^ = 0.023), indicating no statistically significant difference between groups over time.

For negative comments (Figure 1f), a significant group × time interaction was found (*F*(1, 98) = 12.39, *p* < 0.001, partial *η* ^2^ = 0.112), indicating distinct trajectories across groups. At week 10, mothers in the singing group produced about five times fewer negative comments than controls (mean difference = 0.043 [95% CI 0.022, 0.063], *F*(1, 98) = 16.49, *p* < 0.001, partial *η* ^2^ = 0.144), whereas no difference was evident at baseline. These findings indicate that the singing sessions helped mothers adopt a less critical and negative communicative style compared with controls.

#### Effects of the M4M singing intervention on maternal communication were sustained six months post-intervention

**Figure 2.**
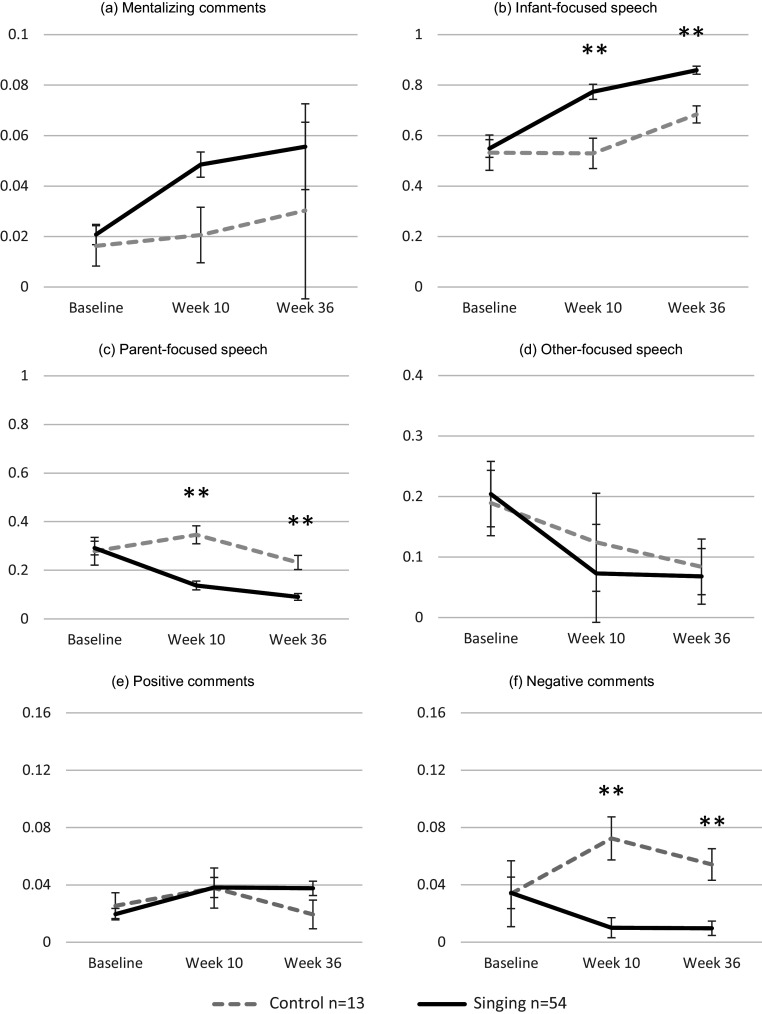
Mean proportion scores for all coded maternal speech domains in the singing and control groups across baseline, week 10, and week 36. Panels (a–f) show mentalizing comments, infant-focused speech, parent-focused speech, other-focused speech, positive comments, and negative comments, respectively (*p < 0.05; **p < 0.01).

Results for differences in the proportions of speech domains between the control and singing groups across the study period (baseline, week 10, and week 36) are presented in Figure 2, with raw data shown for the 67 women who completed video assessments at all three timepoints. Results on main effects of time are reported in the Supplementary Materials (Results).

For mentalizing comments (Figure 2a), there were no significant interaction effects (*F*(2, 65) = 0.273, *p* = 0.644, partial *η* ^2^ = 0.004). However, pairwise comparisons indicated a significant difference between groups at week 10 (*p* = 0.023), but not at week 36 (*p* > 0.05).

For infant-focused speech (Figure 2b), a significant group × time interaction emerged (*F*(2, 65) = 3.86, *p* = 0.02, partial *η* ^2^ = 0.056). Across the study period, mothers in the singing group consistently produced more infant-focused comments than controls, with significant between-group differences at week 36 (mean difference = −0.175 [95% CI –0.250, −0.100], *p* < 0.001).

For parent-focused speech (Figure 2c), a significant group × time interaction was found (*F*(2, 65) = 5.85, *p* = 0.01, partial *η* ^2^ = 0.083). By week 36, mothers in the singing group continued to produce significantly fewer parent-focused comments than controls (mean difference = 0.142 [95% CI 0.076, 0.207], *p* < 0.001). These results indicate that the M4M intervention produced a durable decrease in self-directed or controlling communication, suggesting a lasting shift toward more reciprocal and infant-centered exchanges.

For other-focused speech (Figure 2d), there was no significant group × time interaction (*F*(2, 65) = 0.201, *p* = 0.724, partial *η* ^2^ = 0.003), indicating that changes over time did not differ between the singing and control groups.

For positive comments (Figure 2e), the group × time interaction was also nonsignificant (*F*(2, 65) = 1.597, *p* = 0.208, partial *η* ^2^ = 0.024), showing that both groups followed comparable trajectories across timepoints.

For negative comments (Figure 2f), a significant group × time interaction was detected (*F*(2, 65) = 5.02, *p* = 0.01, partial *η* ^2^ = 0.061). Mothers in the singing group produced fewer negative comments than controls, with significant differences maintained at week 36 (mean difference = 0.045 [95% CI 0.020, 0.069], *p* < 0.001). These findings indicate that the reduction in negative or critical speech observed at the end of the M4M intervention was sustained over time, reflecting a stable shift toward a more positive communicative tone.

#### Maternal perception of attachment toward their infant improved in the singing group, but no differences between singing and control groups were observed across the study

**Figure 3.**
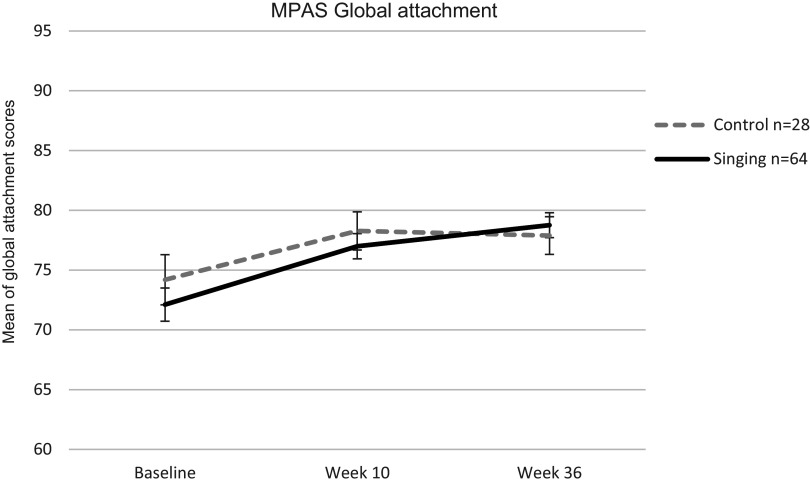
Mean of Global Attachment MPAS scores in the control and singing groups at baseline, week 10, and week 36. *p < 0.05, **p < 0.01.

Results for differences in global MPAS (perceived attachment) scores between the control and singing groups across the study (baseline, week 10, and week 36) are presented in Figure 3, showing group means with raw data points.

A repeated measures ANOVA with Greenhouse–Geisser correction showed a significant main effect of time (*F*(2, 90) = 19.714, *p* < 0.001, partial *η* ^2^ = 0.180), but no significant time × group interaction (*F*(2, 90) = 1.451, *p* = 0.24, partial *η* ^2^ = 0.016). Follow-up pairwise comparisons indicated no significant differences between groups at any timepoint (all *p*s > 0.05). Importantly, these findings refer to maternal self-reported perceived attachment (MPAS total score) and are conceptually distinct from observed maternal communication behaviors assessed using the PCAMs. However, examination of within-group changes revealed that mothers in the singing group showed steady improvements in global MPAS (perceived attachment) scores, increasing significantly from baseline to week 10 (mean difference = −4.88 [95% CI –7.01, −2.76], *p* < 0.001) and from baseline to week 36 (mean difference = −6.65 [95% CI –9.02, −4.27], *p* < 0.001). Between week 10 and week 36, scores significantly increased (mean difference = −1.98 [95% CI –3.47, −0.48], *p* < 0.001), indicating a continued strengthening of perceived attachment following the M4M intervention.

In contrast, mothers in the control group showed an increase in scores from baseline to week 10 (mean difference = −4.09 [95% CI –6.83, −1.35], *p* = 0.01) and an increase from baseline to week 36 (mean difference − 3.70 [95% CI –6.94, −0.46], *p* = 0.04). However, from week 10 to week 36, attachment scores did not meaningfully change (mean difference = 0.373 [95% CI –1.68, 2.43], *p* = 0.73).

### Variables associated with group differences in maternal communication

We conducted bivariate correlations (see Table 2) between SES and clinical variables (EPDS scores at weeks 20 and 36) and the maternal speech domains in which group differences had emerged. At week 10, these domains included mentalizing comments, infant-focused speech, parent-focused speech, and negative speech; at week 36, infant-focused, parent-focused, and negative speech were examined.

**Table 2.** Correlations between clinical and sociodemographic variables and maternal speech at week 10 and week 36. Spearman’s correlation coefficients are presented. (*p < 0.05, **p < 0.01)

	Week 10 speech variables				Week 36 speech variables		
	Mentalizing comments	Infant-focused speech	Parent-focused speech	Negative comments	Infant-focused speech	Parent-focused speech	Negative comments
SES score	.190	.198*	−.227*	−.298*	.211	− .130	− .142
EPDS score at week 20	− .064	− .081	.219*	.021	− .16	.023	− .058
EPDS score at week 36	.051	− .005	.108	.158	− 243	.189	− .097

At week 10, higher SES was significantly associated with more adaptive communicative patterns, showing a positive correlation with infant-focused speech (*r* = 0.198) and negative correlations with parent-focused (*r* = −0.227) and negative speech (*r* = −0.298). EPDS scores assessed at week 20 were not associated with most speech domains but showed a significant positive correlation with parent-focused speech measured at week 10 (*r* = 0.219), indicating that mothers who displayed more parent-focused communication right at the end of the intervention tended to report higher depressive symptoms at the following assessment, approximately two months later. No significant associations were found between EPDS scores at week 36 and any speech domain (all *p*s > 0.05).

No significant correlations were observed for any speech variables at week 36 (all *p*s > 0.05). Taken together, these findings indicate that maternal SES was positively related to the quality of early communicative patterns, and that mothers who used less parent-focused communication at week 10 tended to report lower depressive symptoms at the subsequent assessment. These associations should, however, be interpreted cautiously given the correlational and non-longitudinal nature of the analyses.

## Discussion

This study investigated the impact of the M4M singing intervention on maternal communication and self-reported attachment among mothers with PND. We demonstrate that participation in singing significantly enhanced infant-focused and mentalizing communication and reduced parent-focused and negative speech by week 10, with effects largely sustained through six-month follow-up. By contrast, maternal self-reported attachment, as measured by the MPAS Global Attachment score, improved across both groups, with no significant between-group differences, although gains in the singing group appeared more stable over time. We also found exploratory correlational evidence indicating that higher levels of parent-focused communication at week 10 were positively associated with EPDS scores at week 20, suggesting a possible temporal association between communicative style and subsequent depressive symptoms.

We show that mothers who engaged in singing with their infants were more likely to use infant-focused language, comment on their infants’ internal states, while also reducing speech directed toward their own perspective and employ less negative communicative tones compared with the control group. This pattern suggests an increased ability to support joint attention, affective attunement, and sensitivity to the infant’s experience. Previous research has shown that mothers with PND are less likely to focus on their infant’s mental states, to comment on their experience, and more likely to use intrusive or negatively valenced speech (Murray, Kempton, Woolgar, & Hooper, 1993; Pawlby et al., 2010). Our findings indicate that singing may mitigate these difficulties, strengthening the communicative building blocks of early attachment. Although no statistically significant group differences were observed for positive comments, descriptive trends suggested that mothers in the singing group used slightly more affectively warm expressions. It is possible that singing may foster a more emotionally supportive tone through shared rhythmic engagement and positive affect, though this warrants further investigation in larger samples.

Although global MPAS (perceived attachment) scores improved in both groups, gains appeared more sustained in the intervention arm, raising the possibility that singing may help consolidate maternal perceptions of bonding beyond treatment. Sustained reductions in depressive symptoms in the singing group may have contributed to this pattern, although this was not directly tested statistically. The absence of significant between-group differences in the perceived attachment suggests that this may also reflect broader processes, including time, mood recovery, or engagement in alternative activities.

Beyond the immediate communicative changes observed, it is important to consider the mechanisms through which singing may exert its effects. Singing provides a highly structured yet flexible form of interaction that scaffolds mothers’ ability to engage their infants. Musical rhythm and melody create predictable patterns that can capture the infant’s attention, regulate arousal, and facilitate synchrony, which in turn may encourage mothers to respond in more infant-focused and mentalizing ways (Cirelli, Jurewicz, & Trehub, 2020; Trainor & Cirelli, 2015). From the maternal perspective, singing may also reduce self-consciousness and create a playful, less demanding space for interaction, thereby lowering cognitive load and enabling greater sensitivity to the infant’s cues. Importantly, singing has been shown to directly influence maternal physiology by reducing stress and modulating positive affect and perceived emotional closeness, processes that are known to underpin parental attunement and bonding (Fancourt & Perkins, 2018).

Furthermore, the benefits of the intervention may extend beyond the group sessions themselves. Mothers often continue to use singing spontaneously in daily routines as a way to soothe, engage, or communicate with their infants. Notably, participants in M4M are also given access to a dedicated online song library, allowing them to revisit and use the songs with their babies between sessions and after the program has ended, thereby encouraging continued engagement. Such ongoing use may help consolidate the skills acquired during the intervention, such as sensitivity to the infant’s cues, use of rhythm and melody to regulate affect, and confidence in playful, reciprocal exchanges. By transforming singing into an accessible, everyday tool for emotional regulation and connection, mothers may sustain improvements in communicative attunement and mood well beyond the treatment period. In contrast, control participants were signposted to externally delivered community activities that were not structured as a cohesive intervention, and continued engagement was not systematically monitored. As such, differences in structure and continuity of participation may have contributed to the sustained effects observed in the singing group.

The correlation analyses offer further insight into the relationship between maternal communication and depressive symptoms. In particular, higher levels of parent-focused communication at week 10 were associated with higher EPDS scores at week 20, suggesting a possible link between less adaptive communicative styles and later depressive symptomatology. Given that mothers in the singing group reported significantly lower depressive symptoms at week 20 in the main SHAPER-PND trial (Bind et al., 2025), it is plausible that the observed improvements in parent-focused speech map the same trajectory of improvement of depressive symptoms, or potentially contribute to it mechanistically. This interpretation aligns with evidence that depressive symptoms interfere with attentional flexibility and emotional availability, constraining mothers’ capacity to mentalize and sustain joint attention with their infants (Forman et al., 2007; Stein et al., 2014), and thus the therapeutic success of the M4M singing intervention could lead to a parallel improvement in both dimensions with a more rapid effect on mentalization and a subsequent consolidation on the effects on mood. Higher sociodemographic and socioeconomic status (SES) was associated with more adaptive maternal communication, consistent with prior research linking socioeconomic resources to parenting behaviors. Importantly, intervention-related improvements were observed across the sample, suggesting that structured, arts-based group activities such as singing may support maternal engagement across diverse socioeconomic contexts. Rather than implying protection against structural disadvantage, these findings indicate that accessible group singing may offer relational benefits beyond higher-SES populations. This interpretation aligns with evidence that community-based arts interventions can promote social inclusion and reduce health inequalities (Bungay & Clift, 2010; Daykin et al., 2018).

Several psychotherapeutic approaches have demonstrated benefits in improving maternal sensitivity, communication, and mentalization, including parent–infant psychotherapy, video-feedback interventions, and mentalization-based treatments (Bakermans-Kranenburg, Van IJzendoorn, & Juffer, 2003; Byrne, Murphy, & Connon, 2020; Rebecchini, Bind, & Pariante, 2022; Toth, Rogosch, Manly, & Cicchetti, 2006). However, these therapies are typically resource-intensive, require specialized clinicians, and often reach only a small proportion of mothers in need. In contrast, singing-based interventions represent a low-cost, scalable, and non-stigmatizing alternative that can be delivered in community or primary-care settings by trained facilitators rather than mental health professionals (Bind et al., 2025). Group singing simultaneously promotes emotional regulation and social connectedness, offering a multidimensional pathway to recovery that integrates both psychological and physiological processes. Moreover, because singing is an inherently enjoyable and culturally universal activity, it may attract mothers who might not otherwise seek formal mental health care, thus increasing engagement and adherence. Creative arts interventions may also offer culturally sensitive and accessible forms of support for mothers who might not otherwise engage with formal mental health services. Emerging evidence suggests that such approaches can enhance engagement among diverse and underserved populations, potentially reducing barriers to care (Applewhite et al., 2025; Delattre et al., 2024).

There are several limitations to note. First, the sample size with complete observational data was smaller than that of the full trial, which may have reduced statistical power to detect differences in attachment outcomes. However, comparisons of sociodemographic and clinical characteristics between participants included in the present analyses and those without available data (see Supplementary Table S1) indicated no significant differences, suggesting that the analytic sample was broadly representative of the full cohort. Importantly, our most robust findings were supported by stringent group × time interaction effects. The 2:1 allocation ratio followed the overarching SHAPER-PND hybrid effectiveness–implementation design (Bind et al., 2025; Estevao et al., 2021a), which, for ethical and clinical reasons, aimed to maximize exposure to a promising intervention while retaining a comparison group. Although unequal allocation slightly reduces statistical power, the efficiency loss is modest with moderate ratios. However, by week 36, differential attrition led to smaller control numbers, reducing precision of long-term estimates. Accordingly, sustained effects at follow-up should be interpreted cautiously and require replication in larger samples with more balanced retention.

Second, while the MPAS provided valuable insight into perceived attachment, it is a self-report scale and may not fully capture behavioral dimensions of bonding. Third, the correlational analyses examining associations between communication variables, SES, and depressive symptoms were exploratory and cross-sectional, and therefore do not allow for conclusions regarding directionality or causality. More comprehensive approaches, such as mediation models, would be required to clarify potential pathways linking changes in communication and maternal mood, though these was not feasible here due to sample size constraints.

A further limitation is that session attendance was not analyzed as a dose–response variable. Although retention in the study was higher in the singing group than in the control group (Bind et al., 2025), we cannot determine whether greater exposure was associated with improved outcomes in the mother–infant communication described here, and attendance at control activities was not systematically recorded. Thus, the study evaluates allocation rather than verified exposure levels. Future trials should include systematic attendance tracking and dose–response analyses, especially in relationship with mother–infant communication outcomes.

Additional sources of bias should be acknowledged. Participants knew they were randomized to a singing program or another community activity, so expectancy effects may have operated in both groups, although singing was never presented as having a putative effect on mother–infant communication. Video-recorded interactions may also have introduced reactivity (e.g. more positive or fewer negative comments), but procedures were identical across groups and timepoints, reducing differential bias. As allocation was not blinded, performance bias cannot be excluded.

Finally, infant behavioral outcomes were not directly assessed, and implications for child socioemotional development therefore remain to be determined. Nevertheless, it is noteworthy that ongoing analyses on maternal and infant cortisol in this same sample show that more positive maternal communication (i.e. greater infant-focused attention and lower inattentive engagement) is associated with closer mother–infant cortisol synchrony (Kirkpatrick, et al., under review).

In conclusion, this study is the first to provide observational evidence that a group singing intervention (M4M) can directly enhance maternal communication in mothers with PND, with benefits sustained up to six months and paralleling the improvement in depressive symptoms. These findings highlight the potential of community-based arts interventions to support maternal recovery and strengthen mother–infant relational health. Given the established importance of early interactions for child development, singing-based programs may represent a valuable complement to standard perinatal care, warranting wider implementation and further investigation into long-term effects on both mothers and their children.

## Supporting information

10.1017/S0033291726103997.sm001 Rebecchini et al. supplementary material Rebecchini et al. supplementary material

## Data Availability

The data supporting this study’s findings will be available upon reasonable request from the corresponding author, Lavinia Rebecchini.

## Supplementary material

The supplementary material for this article can be found at http://doi.org/10.1017/S0033291726103997.
