# Supplementary material for: Maternal communication and attachment following a group singing intervention for postnatal depression: findings from the SHAPER-PND trial
Source: Psychol Med. 2026 Apr 7;56:e98. doi: 10.1017/S0033291726103997 (PMC13079224; doi:10.1017/S0033291726103997)
Supplement: Rebecchini et al. supplementary material [file S0033291726103997sup001.docx]

**SUPPLEMENTARY MATERIALS**

**METHODS**

| **Supplementary Figure S1.** SHAPER-PND study participant flow chart. |
| --- |

Enrolled and randomised into trial (n = 199)

Randomised to control & eligibility EPDS completed

n = 66

Randomised to singing & eligibility EPDS completed

n = 133

Consented to video recording n = 185 (93% of total sample)

Singing = 122

Control = 63

Video recorded at baseline n = 160 (86% of participants who consented to video recording)

Singing = 112

Control = 48

Excluded n = 85 (45% of participants who consent to video recording)

Singing = 51

Control = 34

Reason for exclusion:

- Excluded from main outcome analysis n= 22 (singing=15/control=7)
- Unable to reach participant for video recording = 50 (singing n=25, control n=25)
- Baby sleeping n= 1 (singing =1/control=0)
- Poor quality of sound n=4 (singing =4/control=0)
- Video too short n=1 (singing =1/control=0)
- Foreign language n= 4 (singing =4/control=0)
- Twins= 4 (singing =2/control=2)

Video recorded at week 10

n = 120 (65% of participants who consented to video recording)

Singing = 88

Control = 32

Participant included in analysis with both baseline and week 10 videos

n = 100 (54% of participants who consented to video recording)

Singing = 70

Control = 30

Excluded n = 23 (25.5% of participants with video recorded at wk36)

Singing = 18

Control = 5

Reason for exclusion:

- Excluded from main outcome analysis n= 4 (singing =4/control=0)
- I out of screen for most time n=2 (singing =1/control=1)
- Poor quality of sound n=4 (singing =4/control=0)
- Foreign language n= 7(singing =6 /control=1)
- Twins=2 (singing =1/control=1)
- Only baseline and week 36 recorded n=2 (singing =1/control=1)
- Only week 36 video recorded= 2 (singing =1/control=1)

Video recorded at week 36

n = 90 (49% of participants who consented to video recording)

Singing = 72

Control = 18

Participant included in analysis with baseline, week 10 and week 36 videos

n = 67 (36% of participants who consented to video recording)

Singing = 54

Control = 13

| **Supplementary Table S1.**  Sociodemographic, clinical, and infant characteristics of participants included in the present study with complete data (available video recordings) and those without available video data. |
| --- |

|  | Participants included in the present study (n=95-100) | Participants with no data available for the present study (n=87-99) | Statistic |
| --- | --- | --- | --- |
| Maternal age in years, M (SD) | 35.3 (4.2) | 35.6 (5.0) | p = 0.72 |
| Maternal ethnicity, n (%)  Any white background  Ethnic minority groups | 71 (71%)  29 (29%) | 53 (57%)  40 (43%) | **p = 0.04** |
| Maternal qualifications, n (%)  Higher education or above  GCSE/A -level or below | 90 (90%)  10 (10%) | 74 (80.4%)  18 (19.6%) | p = 0.06 |
| Maternal employment status, n (%)  Employed/student/maternity leave  Unemployed/full-time mother | 90 (90%)  10 (10%) | 80 (86%)  13 (14%) | p=0.39 |
| Household income, n (%)  <£30,000  >£30,000 | 79 (83.2%)  16 (16.8%) | 74 (85.1%)  13 (14.9%) | p = 0.73 |
| Maternal marital status, n (%)  Married/cohabiting  Single with/without a partner | 81(81%)  19 (19%) | 79 (84.9%)  14 (15.1%) | p = 0.47 |
| SES score, M (SD) | 0.73 (0.2) | 0.76 (0.2) | p = 0.48 |
| Infant sex, n (%)  Female  Male | 46 (46.5%)  53 (53.5%) | 44 (47.8%)  48 (52.2%) | p = 0.85 |
| Attending other mother-baby group,  n (%)  Yes  No | 71 (71%)  29 (29%) | 55 (59.8%)  37 (40.2%) | p = 0.10 |
| Currently receiving any type of psychological therapy, n (%)  Yes  No | 35 (35%)  65 (65%) | 24 (25.8%)  69 (74.2%) | p = 0.17 |
| Infant age at baseline in months, M (SD) | 4.1 (2.4) | - |  |
| Infant age at week 10 in months, M (SD) | 6.7 (2.5) | - |  |
| Infant age at week 36 in months, M (SD) | 12.4 (2.5) | - |  |
| EPDS baseline score, M (SD) | 16.4 (3.9) | 16.6 (3.8) | p = 0.72 |
| EPDS week 10 score, M (SD) | 10.4 (4.5) | 12.8 (4.9) | **p < 0.001** |
| EPDS week 36 score, M (SD) | 8.8 (4.7) | 11.8 (5.6) | **p < 0.001** |
| Group differences tested with independent-samples t-tests for continuous variables and χ² tests for categorical variables. Minor variations in n reflect missing data. | | | |

**Study design and participants**

Reasons for exclusion are detailed in Figure S1 in the supplementary material. Comparisons between participants included in the present study and those with no available video data are reported in Supplementary Table S1. Overall, the two samples were broadly comparable across sociodemographic and clinical characteristics; however, included participants were more likely to identify as white (71%) than those without available video data (57%) (p = 0.04). In addition, mothers included in the analyses had lower EPDS scores at week 10 (10.4 ± 4.5) and week 36 (8.8 ± 4.7) than those with video data not available (12.8 ± 4.9 at week 10, and 11.8 ± 5.6 at week 36) (all p < 0.001). These differences suggest that although the analytic sample was generally representative of the full cohort, participants who completed the video recordings tended to have slightly lower symptom severity and were less ethnically diverse.

**Randomisation and procedures**

Participants were randomised at a 2:1 ratio (intervention:control), stratified by baseline EPDS score (10-13, 14-19, ≥20) and infant age (0-3 months, 3-6, 6-9) using Sealed Envelope v1.23.1 (Sealed Envelope, n.d.). Assessments were conducted at baseline, week 10 (end of intervention), and week 36. Video recordings were collected through secure Zoom sessions and stored in encrypted databases. Participants also completed questionnaires via REDCap (Harris et al., 2019).

Groups comprised 8–12 mothers and their infants, and sessions were led by a Breathe-trained creative health music lead together with a staff member trained in safeguarding. Mothers and infants sat together in a circle on mats and cushions. Sessions began with welcome songs and icebreakers, followed by the teaching of a variety of songs from around the world, sung in rounds with harmonies. Simple percussion instruments such as maracas and drums were available for mothers and infants to play together, supporting interactive engagement. Weekly reminders via text or phone were used to encourage attendance.

**Measures**

**Sociodemographic and clinical measures**

Sociodemographic and socioeconomic (SES) variables were ascertained with a semi-structured interview. To account for the combined influence of sociodemographic risk factors, a composite socioeconomic status (SES) score was created, following approaches adopted in previous research examining maternal mental health and mother–infant interaction (Bind et al., 2021; Rebecchini et al., 2023), and in line with evidence supporting cumulative risk models in child development (Evans et al., 2013). It encompassed maternal age, ethnicity (white vs. ethnic minority), marital status (married/cohabiting vs. single with or without partner), occupation (employed/student vs. unemployed or full-time mother), and educational attainment (A-level or higher vs. GCSE or lower), all assessed at baseline, with lower scores indicating higher vulnerability.

*Maternal perceived bonding towards the infant*

Perceived maternal attachment was assessed at baseline, week 10, and week 36 using the Maternal Postnatal Attachment Scale (MPAS; Condon & Corkindale, 1998)). The MPAS is a 19-item self-report questionnaire designed to evaluate the emotional bond between a mother and her infant in the early postnatal period. It comprises four subscales: Quality of Attachment, Pleasure in Interaction, and Absence of Hostility, as well as a Global Attachment score. Items are rated on a 5-point Likert scale, and higher scores indicate stronger attachment. The total score ranges from 19 to 95.

There is no single universally agreed-upon cut-off score for the MPAS (Condon & Corkindale, 1998), as it is a research tool rather than a diagnostic measure. Scores are interpreted along a spectrum, with higher scores reflecting more optimal bonding, but not defining clinical thresholds. Previous studies have highlighted the absence of consensus regarding definitive cut-offs for low attachment (Bird et al., 2022). The MPAS has, however, been widely used in perinatal research and has demonstrated sensitivity to variations in bonding and risk for psychopathology (Biaggi et al., 2016).

Given that the three of the four MPAS subscales (quality of attachment, absence of hostility, and pleasure in interaction) were all highly correlated with the global attachment score (all p < 0.001), only the global attachment measure was included in the present analyses. This score was selected as it provides an overarching index of maternal attachment while minimizing redundancy across subscales.

**PCAMs**

All videos were coded by a primary trained rater (LR). To assess inter-rater reliability, a subset of videos was independently double-coded by two additional trained raters (FM and AK). All coders were blinded to participants’ group allocation and assessment timepoint. Inter-rater reliability was evaluated using intraclass correlation coefficients (ICCs). Across domains, single-measure ICCs ranged from 0.48 to 0.96, indicating moderate to excellent agreement between raters.

(i) *Mentalizing comments.* This category captured maternal utterances that referred to or interpreted the infant’s internal experiences. Four subtypes were coded: (a) internal state agency comments, where mothers spoke on behalf of the infant (e.g., “I’m a happy baby”); (b) cognitive state attributions, reflecting awareness of the infant’s intentions or desires (e.g., “you want that bottle”); (c) emotional state references (e.g., “you like this”); and (d) references to bodily states such as hunger or fatigue (e.g., “you are hungry”). These comments reflected the mother’s capacity to recognize, interpret, and verbalize the infant’s mental and physical states. They represent sensitivity to the infant’s mind and are consistent with the construct of maternal mind-mindedness (Meins & Fernyhough, 1999).

(ii) *Attentional focus of maternal speech.* Mothers’ overall attentional focus in their speech was classified into three domains: infant-focused, parent-focused, and other-focused speech.

- *Infant-focused speech* included utterances directed to or about the infant. This encompassed external state agency comments, such as following the infant’s gaze or actions (e.g., “are you looking at the colours?”; “are you trying to stretch your arms out?”), vocalisations (e.g., repeating “da da da”; “is that a smile?”), descriptions of the infant’s physical features or clothing (e.g., “you have such beautiful eyes”; “you have pink socks”), and attention-maintaining remarks such as greetings and songs.
- *Parent-focused speech* included utterances in which the mother attempted to capture or redirect the infant’s attention toward her own agenda, rather than following the infant’s current play or attentional focus (i.e., absence of joint attention). This category reflected moments where the mother sought to elicit particular responses or behaviours from the infant, often through instructions, corrections, or demands (e.g., “don’t frown at me, smile like you smile at Daddy”). It also included self-referential statements in which the mother commented on her own feelings, abilities, or experiences (e.g., “I’m not able to make you smile”; “Mummy’s not as good as Daddy”). These types of utterances highlight instances where the maternal focus was centred on her own perspective rather than on the infant’s ongoing activity, in line with prior descriptions of maternal speech (Murray et al., 1993).
- *Other-focused speech* encompassed comments unrelated to the immediate interaction, often displaced in time or context (e.g., “I wonder what Daddy is doing”; “you had a long sleep”), as well as remarks about the set-up (e.g., the camera) or expressions of self-consciousness (e.g., “how much longer?”).

(iii) *Affective tone of speech.* Only utterances that conveyed a clear affective quality were coded. Positive affective tone included statements with an affectionate or rewarding connotation, such as praise, endearments, or speech delivered with a warm tone (e.g., “good girl,” “my sweet baby”). Negative affective tone included utterances that were more directive, corrective, or expressed irritation or frustration (e.g., “don’t do that,” “why are you so naughty today”), reflecting a negative or strained emotional quality during communication. Neutral utterances were not classified in this dimension.

**Statistical analysis**

This section provides additional details on assumption testing, corrections, sensitivity analyses, and missing data handling referenced in the main manuscript.

Prior to analyses, data were screened for missingness, outliers, accuracy, and normality. Normality was assessed using the Shapiro–Wilk test and visual inspection of Q–Q plots and histograms. Although some variables showed deviations at specific timepoints, distributions were approximately normal. Given the robustness of repeated measures ANOVA to moderate deviations, parametric analyses were retained. Non-parametric sensitivity analyses (Friedman and Wilcoxon tests) showed comparable results.

Two-way repeated measures ANOVAs examined group (singing vs control) × time (baseline, week 10, week 36) effects for PCAMs domains and MPAS global attachment scores. Mauchly’s test indicated violations of sphericity for mentalizing comments, parent-focused speech, other-focused speech, negative comments, and MPAS global scores (all ps < .01); Greenhouse–Geisser corrections were therefore applied (ε values reported in Results). Sphericity was met for infant-focused and positive comments.

Missing MPAS data were handled using an intention-to-treat approach with last observation carried forward (LOCF), consistent with the main SHAPER-PND analyses (Bind et al., 2025).

Pearson’s correlation coefficients (r) were computed between SES, EPDS scores, and maternal speech domains in which significant group differences had emerged. Consistent with our aim to explore variables sensitive to the intervention, analyses were restricted to communication domains demonstrating group effects; MPAS (perceived attachment) scores were not included, as no significant between-group differences were observed.

**SUPPLEMENTARY RESULTS**

*Maternal communication was significantly greater in the singing intervention group compared with the control group at the end of the intervention*

Results for main effects of time across all coded maternal speech domains are presented below.

For mentalizing comments, there was a significant main effect of time (F(1, 98) = 6.888, p = 0.010, partial η² = 0.066), indicating an overall enhancement in maternal mentalization over time. Post hoc comparisons showed that this increase was primarily driven by the singing group, where the proportion of mentalizing comments was approximately 2.2 times higher at week 10 than at baseline (mean difference = –0.02976 [95% CI –0.042, –0.018], F(1, 98) = 28.60, p < 0.001, partial η² = 0.226). In contrast, no significant change was observed in the control group (p = 0.717).

For infant-focused speech, a significant main effect of time was also found (F(1, 98) = 12.939, p < 0.001, partial η² = 0.117), reflecting a general increase in infant-oriented communication. Post hoc analyses indicated that this improvement was largely driven by the singing group, where proportions of infant-focused speech were approximately 1.4 times higher at week 10 than at baseline (mean difference = –0.241 [95% CI –0.311, –0.171], F(1, 98) = 51.93, p < 0.001, partial η² = 0.346). No meaningful change was detected in the control group (p = 0.667).

For parent-focused speech, there was a significant main effect of time (F(1, 98) = 6.344, p = 0.013, partial η² = 0.061), showing an overall reduction across the sample. This decline was primarily driven by the singing group, where parent-focused speech was roughly 2.2 times lower at week 10 than at baseline (mean difference = 0.174 [95% CI 0.117, 0.230], F(1, 98) = 42.40, p < 0.001, partial η² = 0.302). In contrast, the control group showed no meaningful change (p = 0.213).

For other-focused speech, there was a significant main effect of time (F(1, 98) = 13.010, p < 0.001, partial η² = 0.117), indicating an overall reduction in comments unrelated to the play context. Within-group analyses showed that this decrease was mainly driven by the singing group, where other-focused speech was approximately 2.3 times lower at week 10 than at baseline (mean difference = 0.083 [95% CI 0.039, –0.126], F(1, 98) = 16.17, p < 0.001, partial η² = 0.142). The control group showed a smaller, nonsignificant reduction (p = 0.096). The group × time interaction was nonsignificant (F(1, 98) = 0.636, p = 0.427, partial η² = 0.006), suggesting parallel decreases across groups.

For positive comments, there was no significant main effect of time (F(1, 98) = 2.892, p = 0.133, partial η² = 0.023). However, within-group comparisons indicated a significant increase in the singing group, where positive comments were approximately 1.5 times higher at week 10 than at baseline (mean difference = –0.015 [95% CI –0.026, –0.005], F(1, 98) = 8.61, p = 0.004, partial η² = 0.081). No changes were observed in the control group (p = 0.911). This suggests that the singing intervention selectively promoted a more positive and affectively enriched tone of maternal speech.

For negative comments, no overall main effect of time was observed (F(1, 98) = 0.090, p = 0.765, partial η² = 0.001), indicating that overall frequencies of negative speech remained stable. Within-group analyses, however, revealed divergent trajectories: mothers in the singing group showed a marked reduction in negative comments from baseline to week 10 (F(1, 98) = 8.64, p = 0.004, partial η² = 0.081), whereas controls showed a small but significant increase (F(1, 93) = 5.21, p = 0.025, partial η² = 0.050).

*Effects of the singing intervention on maternal communication were sustained six months post-intervention*

Results for main effects of time across all coded maternal speech domains are presented below.

For mentalizing comments, there was no significant main effect of time (F(2, 65) = 1.03, p = 0.327, partial η² = 0.016). However, within-group analyses indicated that, in the singing group, mentalizing comments were somewhat higher at week 36 than at baseline, although this increase did not reach significance (mean difference = –0.035 [95% CI –0.073, 0.003], p = 0.073). These findings suggest that mothers maintained previously gained improvements in reflective communication over time, rather than showing additional increases.

For infant-focused speech, a significant main effect of time emerged (F(2, 65) = 15.14, p < 0.001, partial η² = 0.189). Across the three timepoints, mothers in the singing group consistently produced more infant-focused comments than controls, with significant group differences at week 36 (mean difference = –0.175 [95% CI –0.250, –0.100], p < 0.001). Within-group analyses revealed further increases in both groups from week 10 to week 36 (singing mean difference = –0.086 [95% CI –0.152, –0.019], p < 0.001; control mean difference = –0.154 [95% CI –0.275, –0.033], p = 0.050). These results indicate continued growth in infant-oriented speech, suggesting that mothers became increasingly responsive and attuned to their infants’ cues over the follow-up period.

For parent-focused speech, analyses revealed a significant main effect of time (F(2, 65) = 7.12, p = 0.003, partial η² = 0.099). By week 36, mothers in the singing group continued to produce markedly fewer parent-focused comments than controls (mean difference = 0.142 [95% CI 0.076, 0.207], p < 0.001). Within-group comparisons showed that parent-focused speech decreased significantly from baseline to week 36 (mean difference = 0.201 [95% CI 0.141, –0.261], p < 0.001) and from week 10 to week 36 (mean difference = 0.047 [95% CI 0.005, –0.089], p = 0.03) in the singing group, while the control group showed a smaller decline only between week 10 and week 36 (mean difference = 0.114 [95% CI 0.012, –0.215], p = 0.03). These findings indicate a sustained reduction in self-referential or directive communication, suggesting that the intervention produced long-term improvements in mothers’ ability to remain focused on and responsive to their infants.

For other-focused speech, there was a significant main effect of time (F(2, 65) = 9.34, p = 0.001, partial η² = 0.126). Both groups showed reductions in other-focused speech at week 36 compared with baseline (singing mean difference = 0.241 [95% CI 0.183, –0.299], p < 0.001; control mean difference = 0.194 [95% CI 0.052, –0.337], p = 0.05). This pattern reflects a general decline in off-topic comments across time, suggesting that both groups became more absorbed in the interaction and better able to maintain shared focus with their infants.

For positive comments, there was no significant main effect of time (F(2, 65) = 2.49, p = 0.09, partial η² = 0.037). However, within-group analyses showed that positive comments increased in the singing group from baseline to week 36 (mean difference = –0.018 [95% CI –0.029, –0.007], p = 0.001), while controls remained stable (p > 0.05). This suggests that the intervention fostered enduring improvements in mothers’ affective expression, promoting a more encouraging and emotionally warm communicative tone over time.

For negative comments, there was no significant main effect of time (F(2, 65) = 0.38, p = 0.65, partial η² = 0.006), but within-group comparisons showed a significant decrease in negative comments from baseline to week 36 in the singing group (mean difference = 0.025 [95% CI 0.001, –0.048], p = 0.041), while no change was found among controls (p > 0.05). These results indicate that mothers who participated in the singing sessions maintained their reduced use of negative or critical speech, reflecting a stable and positive shift in communicative tone.

REFERENCES

Bind, R. H., Lawrence, A. J., Estevao, C., Hazelgrove, K., Priestley, K., Rebecchini, L., Laijawala, R., Miller, C., Healey, A., Agwuna, J., Sevdalis, N., Bakolis, I., Davis, R., Lopez, M. B., Woods, A. J., Crane, N., Manoharan, M., Burton, A., Dye, H., … Pariante, C. M. (2025). Clinical effectiveness, implementation effectiveness and cost-effectiveness of a community singing intervention for postnatal depressive symptoms, SHAPER-PND: randomised controlled trial. *The British Journal of Psychiatry*, 1–10. https://doi.org/10.1192/bjp.2025.10377

Condon, J. T., & Corkindale, C. J. (1998). The assessment of parent-to-infant attachment: Development of a self-report questionnaire instrument. *Journal of Reproductive and Infant Psychology*, *16*(1), 57–76. https://doi.org/10.1080/02646839808404558

Harris, P. A., Taylor, R., Minor, B. L., Elliott, V., Fernandez, M., O’Neal, L., McLeod, L., Delacqua, G., Delacqua, F., & Kirby, J. (2019). The REDCap consortium: building an international community of software platform partners. *Journal of Biomedical Informatics*, *95*, 103208.

Meins, E., & Fernyhough, C. (1999). Linguistic acquisitional style and mentalising development: The role of maternal mind-mindedness. *Cognitive Development*, *14*(3), 363–380.

Murray, L., Kempton, C., Woolgar, M., & Hooper, R. (1993). Depressed Mothers’ Speech to Their Infants and its Relation to Infant Gender and Cognitive Development. In *J. Child FiKhai Piyckat* (Vol. 34, Number 7).

Sealed Envelope. (n.d.). *https://www.sealedenvelope.com/simple-randomiser/v1/*. Https://Www.Sealedenvelope.Com/Simple-Randomiser/V1/.
